# Changes in Cardiac Varices and Their Clinical Significance after Eradication of Esophageal Varices by Band Ligation

**DOI:** 10.1155/2016/2198163

**Published:** 2016-12-26

**Authors:** Seung Woon Park, Yeon Seok Seo, Han Ah Lee, Sang Jung Park, Tae Hyung Kim, Jae Min Lee, Sang Jun Suh, Hyuk Soon Choi, Eun Sun Kim, Bora Keum, Young Kul Jung, Ji Hoon Kim, Hyonggin An, Hyung Joon Yim, Yoon Tae Jeen, Jong Eun Yeon, Hong Sik Lee, Hoon Jai Chun, Kwan Soo Byun, Soon Ho Um, Chang Duck Kim

**Affiliations:** ^1^Department of Internal Medicine, Korea University College of Medicine, Seoul, Republic of Korea; ^2^Department of Biostatistics, Korea University College of Medicine, Seoul, Republic of Korea

## Abstract

*Background and Aims*. Cardiac varices (CVs) in patients with type 1 gastroesophageal varices (GOV1s) usually disappear with treatment for esophageal varices (EVs) by endoscopic injection sclerotherapy (EIS). However, whether this applies to patients treated with endoscopic band ligation (EBL) for EVs remains unclear. We evaluated the effect of EVs eradication by EBL on CVs.* Methods*. We included cirrhotic patients whose EVs had been eradicated using EBL and excluded those who had been treated using EIS, those who had received endoscopic therapy for CVs, and those who were combined with hepatocellular carcinoma.* Results*. A total of 123 patients were enrolled. The age was 59.7 ± 11.7 years, and 96 patients (78.0%) were men. Thirty-eight patients (30.9%) had EVs only, while 85 (69.1%) had GOV1s. After EVs eradication, the CVs disappeared in 55 patients (64.7%). EVs recurred in 40 patients, with recurrence rates at 1, 2, and 3 years of 16.0%, 29.6%, and 35.6%, respectively, the recurrence being more frequent in patients who had undergone EBL for secondary prophylaxis and in those with persisting CVs after EVs eradication (*P* = 0.003).* Conclusions*. CVs frequently disappeared when EVs were eradicated using EBL in patients with GOV1s. Persistence of CVs after EVs eradication by EBL was associated with EVs recurrence.

## 1. Introduction

Bleeding from gastroesophageal varices (GOVs) is a common and severe adverse event during liver cirrhosis, presenting in approximately 50% of patients with cirrhosis [1]. Once varices develop, they frequently bleed in 10–15% of the year [2]. Although the prognosis for patients with variceal bleeding has improved significantly during the last few decades, the condition remains fatal in about 15–20% of cases [3–5]. In addition, rebleeding after hemostasis is very common without appropriate prophylactic treatments [3, 6]. Because of the poor prognosis in case of bleeding from GOVs, most practice guidelines recommend prophylactic treatment in patients with a high risk of bleeding [7–9].

Nowadays, because of the superiority to endoscopic injection sclerotherapy (EIS) on the efficacy and safety, endoscopic band ligation (EBL) is considered the first-choice treatment for hemostasis and prophylaxis of bleeding from esophageal varices (EVs) [7–10]. However, although EVs can be easily eradicated using repeated EBL, EVs recurrence after eradication is common. Furthermore, the recurrence rate after EBL is significantly higher than that after EIS [9, 11], probably because the two procedures differ in their mechanism of action. In EBL, EVs are eradicated through mechanical strangulation; this effect is usually limited to the mucosa and submucosa, while perforating veins between the paraesophageal veins and submucosal veins are preserved. In contrast, EIS eradicates EVs through a chemical reaction that leads to fibrosis; the effects of EIS extend into the deeper layers, obliterating the perforating veins. EVs recurrence is associated with an increased risk of bleeding, and it is therefore crucial that patients be monitored closely after treatment for EVs.

In many patients, EVs coexist with gastric varices (GVs), which are usually sorted according to Sarin's classification, into the four following categories: type 1 and type 2 GOVs (GOV1s and GOV2s) and type 1 and type 2 isolated GVs (IGV1s and IGV2s) [12]. GOV1s extend below the gastroesophageal junction along the lesser curvature of the stomach, while GOV2s extend below the gastroesophageal junction into the fundus [12]. In 20–60% of patients, GVs of these types disappear within 6 months of EIS treatment [12, 13], probably because sclerosant flows in a caudal direction towards the GVs [14–16]. For this reason, it is recommended that patients with GOVs should have their EVs treated first; after 6 months, if the GVs persist, then specific therapy for the GVs should be considered if indicated [17]. However, it is now unclear whether this recommendation can be applied, since EBL, rather than EIS, is used to eradicate EVs. In addition, cardiac varices are thought to act as feeding pathways for EVs, and several studies have suggested that the vascular structure in the cardia is associated with EVs recurrence.

Therefore, this study aimed to evaluate the changes in cardiac varices, as well as their clinical significance, after EVs eradication by EBL in patients with liver cirrhosis and GOV1s.

## 2. Methods

### 2.1. Patients

All consecutive patients who had undergone EVs eradication by EBL were included. Patients who had been treated using EIS were excluded, as were those with history of endoscopic therapy for cardiac varices and those with concomitant hepatocellular carcinoma (HCC) or other malignancy. In addition, patients who had undergone endoscopic treatment for cardiac varices alongside their EBL treatment were also excluded. The protocol was approved by the Human Ethic Committee of the Korea University Anam Hospital and conformed to the ethical guidelines of the 1975 Declaration of Helsinki. A waiver of consent was obtained, and the patient records were anonymized and deidentified prior to analysis.

Patients were classified as having EVs only, GOVs, and IGVs, depending on the presence of accompanying cardiac or fundal varices. Cardiac varices were defined as varices which were continuous with the EVs and extended for 2–5 cm below the gastroesophageal junction, along the lesser or greater curvatures of the stomach. GOVs were further classified according to Sarin's classification as follows: type 1 GOVs (GOV1s) extended below the gastroesophageal junction along the lesser curvature of the stomach and type 2 GOVs (GOV2s) extended below the gastroesophageal junction into the fundus [12].

**Figure 1:**
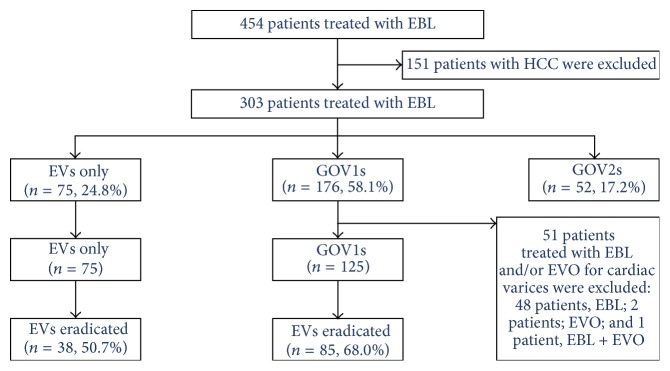
Flow chart of patients who had been treated using endoscopic band ligation. EBL: endoscopic band ligation; EVs: esophageal varices; GOVs: gastroesophageal varices; HCC: hepatocellular carcinoma.

Figure 1 shows a flow chart of patients who underwent EBL at our hospital. Among the 454 patients who underwent EBL, 151 had HCC and were excluded. Among the remaining 303 patients, 75 (24.8%) had EVs only, 176 (58.1%) had GOV1s, and 52 (17.2%) had GOV2s. Among the 176 patients with GOV1s, 51 (29.0%) were excluded because they had also undergone endoscopic treatment for cardiac varices. Among the 200 patients with either EVs only (75 patients) or GOV1 (125 patients), EVs were eradicated in 123 patients (61.5%): 38 of the 75 patients with EVs only (50.7%) and 85 of the 125 patients with GOV1s (68.0%). The main causes of EVs eradication failure were loss to follow-up and patients' refusal of further endoscopy.

### 2.2. Data Collection

All medical records were reviewed to identify patients who had undergone EBL for EVs. Age, sex, and underlying liver disease data were analyzed. The following laboratory investigations were carried out at baseline: platelet count, international normalized ratio (INR), and serum aspartate aminotransferase (AST), alanine aminotransferase (ALT), bilirubin, albumin, creatinine, and sodium levels. The Child-Pugh score was determined by applying Pugh's commonly used modification, which is based on the presence and severity of ascites and hepatic encephalopathy, prolongation of the prothrombin time, and levels of serum bilirubin and albumin [18]. The size of the EVs and cardiac varices was measured at the initial endoscopy—before treatment with EBL; the size of EVs was measured according to Beppu's classification [19]. To measure the size of the cardiac varices, the diameter and scope were measured in the endoscopic pictures that had been taken before endoscopic treatment. The real diameter of the cardiac varices was calculated using the ratio of the measured diameter of the cardiac varices to the measured diameter of the scope, as well as the real diameter of the scope (9.8 mm; GIF-H260; Olympus Optical Co., Ltd., Tokyo, Japan).

EBL sessions were repeated at 4-week intervals until EVs were eradicated. Specifically, EVs were considered eradicated when they had (1) disappeared entirely or (2) decreased in size to ≤grade 1 and were thus too small for EBL and lost the red color sign. The numbers of EBL sessions and used rubber bands, as well as the duration until eradication, were recorded for analysis. After EVs eradication, follow-up endoscopy was performed 1 month later and then at 3-month intervals for 1 year. Follow-up intervals were then prolonged to 6 months when EVs had not recurred during the 1-year follow-up. EVs recurrence was defined as (1) an increase in EV size to ≥grade 2, (2) appearance of the red color sign, or (3) the development of bleeding from EVs.

### 2.3. Statistics

Statistical analyses were performed using the Statistical Package for the Social Science (SPSS) version 20.0 for Windows (SPSS, Inc., Chicago, IL). All data are expressed as either mean ± standard deviation or number of patients (percentage of the entire cohort) for continuous and categorical variables, respectively. Categorical and quantitative variables were compared between the groups by using the chi-square test and Student's* t*-test, respectively. The Kaplan-Meier method was used to estimate the cumulative incidences of EVs recurrence or bleeding. The Cox proportion hazard model was utilized to analyze factors associated with EVs recurrence or bleeding. All tests were two-tailed, and *P* values < 0.05 were considered statistically significant.

## 3. Results

### 3.1. Baseline Characteristics

A total of 123 patients who had undergone EVs eradication by EBL were enrolled in this study.  Table 1 shows the baseline characteristics of the patients according to the type of varices at baseline. The patients' age was 59.7 ± 11.7 years, and 96 patients (78.0%) were men. Alcoholic liver disease was the most common underlying liver disease (59 patients, 48.0%), followed by chronic hepatitis B (41 patients, 33.3%). The Child-Pugh score was 7.2 ± 1.7. EBL was performed as a primary prophylaxis in 71 patients (57.7%) and as a secondary prophylaxis in 52 patients (42.3%). Regarding the size of the EVs, 104 patients (84.6%) had EVs of size F2, while 19 (15.4%) had EVs of size F3. The red color sign was noted in 117 patients (95.1%). With regard to the type of varices at baseline, 38 patients (30.9%) had EVs only, and 85 (69.1%) had GOV1s. The baseline characteristics were comparable between these two groups (Table 1).

### 3.2. EVs Eradication by EBL

**Figure 2:**
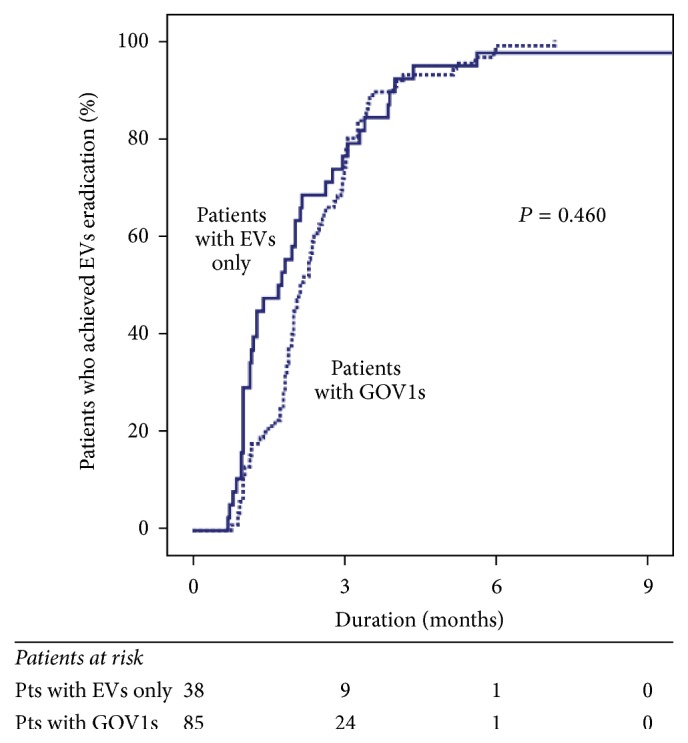
Cumulative incidence for eradication of esophageal varices by endoscopic band ligation—arranged according to the type of varices at baseline. EVs: esophageal varices; GOVs: gastroesophageal varices.

In all enrolled patients, the EVs were eradicated using an average of 7.7 ± 3.6 bands in 2.3 ± 1.0 sessions during 2.4 ± 1.4 months. The numbers of EBL sessions did not differ between patients with EVs only and those with GOV1s (2.1 ± 1.2 sessions versus 2.4 ± 0.8 sessions, resp.; *P* = 0.112); similarly, neither the number of rubber bands used (7.3 ± 5.0 bands versus 7.9 ± 2.7 bands, resp.; *P* = 0.456) nor the duration until eradication (2.2 ± 1.7 months versus 2.5 ± 1.2 months, resp.; *P* = 0.288) differed between the groups. In addition, the cumulative incidence of EVs eradication by EBL did not differ between the two groups (*P* = 0.460) (Figure 2).

### 3.3. Changes in Cardiac Varices after EVs Eradication in Patients with GOV1s

The size of the cardiac varices before EBL was 1.1 ± 0.3 cm in the 85 patients with GOV1s. After EVs eradication, the cardiac varices had disappeared in 55 patients (64.7%), diminished in 20 patients (23.5%), and remained unchanged in 10 patients (11.8%). The disappearance of cardiac varices after EBL was not correlated with the size of the EVs, presence of the red color sign on the EVs at baseline, number of EBL sessions, number of rubber bands used, or duration until EVs eradication (Table 2). Cardiac varices were significantly smaller before EBL in patients whose cardiac varices had disappeared than in those whose cardiac varices had persisted (1.0 ± 0.2 cm versus 1.1 ± 0.3 cm, resp.; *P* = 0.046). Among the 85 patients with GOV1s, 19 (22.4%) had cardiac varices with a diameter of ≤0.9 cm at baseline, while 66 (77.6%) had cardiac varices with a diameter of >0.9 cm. The cardiac varices disappeared more frequently in patients with a baseline cardiac varices diameter of ≤0.9 cm (16 of 19 patients, 84.2%) than in those with a baseline cardiac varices diameter of >0.9 cm (39 of 66 patients, 59.1%; *P* = 0.043).

### 3.4. EVs Recurrence after Eradication

**Figure 3:**
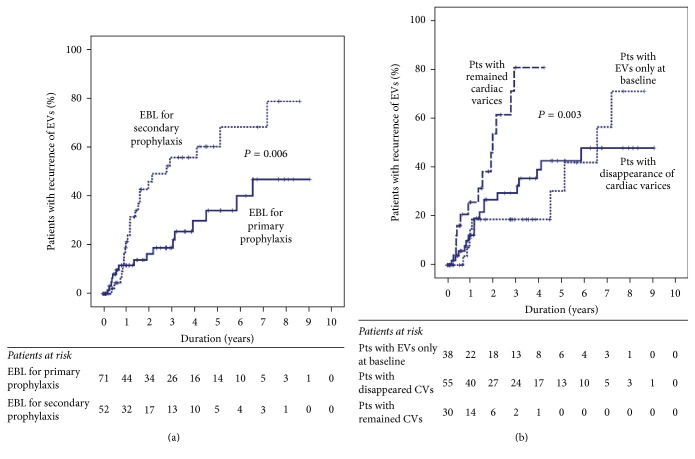
Cumulative incidence of recurrence for esophageal varices after eradication by endoscopic band ligation according to (a) the purpose of endoscopic band ligation and (b) the changes in cardiac varices after eradication of esophageal varices. EBL: endoscopic band ligation; EV: esophageal varices.

During follow-up, EVs recurred in 40 patients, with recurrence rates at 1, 2, and 3 years of 16.0%, 29.6%, and 35.6%, respectively. The EVs recurrence rate was not correlated with age, sex, type of varices at baseline, or Child-Pugh score (Table 3). Furthermore, recurrence was more frequent in patients who had undergone EBL for secondary prophylaxis than in those who had undergone EBL for primary prophylaxis (Figure 3(a)). In addition, the EVs recurrence rate was significantly associated with the extent of change in the cardiac varices after EVs eradication (Figure 3(b)). Specifically, the recurrence rate was significantly higher in patients whose cardiac varices had persisted after EVs eradication than in others (*P* = 0.003), while it was comparable between patients with EVs only and those whose cardiac varices had disappeared after EVs eradication. Both the purpose of EBL (hazard ratio [HR], 2.557; 95% confidence interval [CI], 1.346–4.855; *P* = 0.004) and the extent of change in cardiac varices after EVs eradication (HR, 1.968; 95% CI, 1.240–3.122; *P* = 0.004) were also significantly associated with EVs eradication on multivariate analysis.

### 3.5. Variceal Bleeding after EVs Eradication

**Figure 4:**
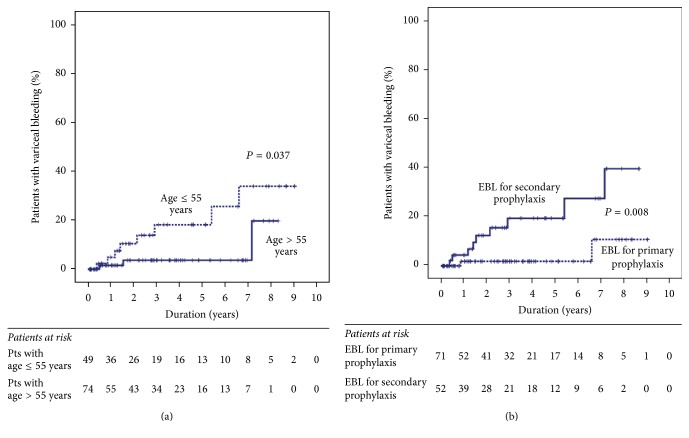
Cumulative incidence of variceal bleeding after eradication of esophageal varices by endoscopic band ligation—arranged according to (a) age and (b) the purpose of endoscopic band ligation. EBL: endoscopic band ligation.

During the follow-up period, EVs bleeding occurred in 11 patients, and the cumulative incidences of variceal bleeding at 1, 2, and 3 years were 2.9%, 6.5%, and 9.5%, respectively. EVs bleeding was significantly associated with age, purpose of EBL, and serum creatinine levels (Table 3); specifically, it was more frequent in younger patients (*P* = 0.037) (Figure 4(a)) and in patients who had undergone EBL for secondary prophylaxis (*P* = 0.008) (Figure 4(b)). Age (HR, 0.910; 95% CI, 0.847–0.978; *P* = 0.010) and purpose of EBL (HR, 6.520; 95% CI, 1.353–31.419; *P* = 0.019) were also significantly associated with variceal bleeding after EVs eradication on multivariate analysis, while serum creatinine level was not (HR, 0.242; 95% CI, 0.025–2.315; *P* = 0.218).

### 3.6. Mortality

During the follow-up period, 12 patients died. The causes of mortality were liver failure in six patients, bacterial infection in five patients, and intracranial hemorrhage in one patient. The cumulative mortality rates at 1, 2, and 3 years were 2.8%, 6.3%, and 9.2%, respectively. Age, Child-Pugh score, and serum sodium level were associated with mortality in the univariate analysis (Table 3). Upon multivariate analysis, age (HR, 1.133; 95% CI, 1.035–1.240; *P* = 0.007) and Child-Pugh score (HR, 2.198; 95% CI, 1.336–3.616; *P* = 0.002) were significantly associated with mortality.

## 4. Discussion

In patients with GOVs, cardiac varices usually disappear when EVs are eradicated using EIS [12, 13] because sclerosant flows in a caudal direction towards the cardiac varices [14–16]. For this reason, practice guidelines do not recommend specific treatment for cardiac varices until EVs have been eradicated using endoscopic therapy [17]. However, because EBL obliterates EVs through a different mechanism, its effects are usually limited to the superficial layers. Therefore, it is unclear whether cardiac varices disappear when EVs are eradicated using EBL.

In this regard, several previous studies have shown conflicting results regarding the influence of EBL on cardiac varices; in one study, cardiac varices disappeared at similar frequencies between EIS (61.5%) and EBL (50%) [9], while another study suggested that cardiac varices do not disappear after EVs eradication by EBL in patients with GOVs [20]. In the present study, cardiac varices had disappeared in 64.7% of patients with GOV1s who had undergone EVs eradication by EBL. This incidence is comparable to that of EVs eradication by EIS in the previous study [9]. Therefore, our study suggests that no specific treatment for cardiac varices is needed in patients with GOV1s who are to undergo EBL for EVs eradication.

Although EVs can be easily eradicated using repeated EBL, the procedure is associated with a high frequency of EVs recurrence [9, 11]. In the present study, EVs recurrence rates at 1 and 2 years after EVs eradication were 16.0% and 29.6%, respectively, corroborating previous studies. Therefore, because EVs recurrence is associated with an increased risk of variceal bleeding and mortality, regular follow-up endoscopy to monitor EVs recurrence is required. In addition, to differentiate patients at high risk of EVs recurrence and provide more careful surveillance to these patients, it would be helpful to determine the risk factors for EVs recurrence after eradication. In this study, the purpose of EBL (primary or secondary prophylaxis) and the change in cardiac varices after EBL were independent predictors of EVs recurrence. Specifically, there was a higher risk of EVs recurrence in patients who had undergone EBL for secondary prophylaxis than in those who had undergone EBL for primary prophylaxis. This was to be expected, given that secondary prophylaxis is performed in patients who had experienced episodes of variceal bleeding, while primary prophylaxis is performed in patients who have never experienced such bleeding. In patients with liver cirrhosis and portal hypertension, submucosal veins in the cardia of the stomach are significantly dilated and communicate directly with EVs via the palisade zone at the gastroesophageal junction. Therefore, cardiac varices are thought to act as feeding vessels for EVs [15]. Several studies have evaluated the relationship between cardiac varices and EVs recurrence by using endoscopic ultrasound; they have suggested that large cardiac varices on endoscopic ultrasound—whether performed before [21] or after [22] EBL—are closely associated with EVs recurrence. Therefore, the incidence of EVs recurrence is significantly lower in patients whose cardiac varices have disappeared with EVs eradication, perhaps because the disappearance of cardiac varices represents the obliteration of perforating veins during EBL. Relatedly, in the present study, cardiac varices disappeared with EVs eradication more frequently in patients whose cardiac varices were smaller at baseline. Therefore, it may be that patients whose cardiac varices disappeared with EVs eradication had a lower incidence of EVs recurrence because cardiac varices disappear more frequently in patients with smaller, less severe varices. Regardless of the mechanism, this study suggested that persistence of cardiac varices after EVs eradication is a risk factor for EVs recurrence; therefore, a more strict surveillance strategy is needed in these patients.

Because GOVs development is thought to be a compensatory mechanism to the increased portal pressure, EVs eradication may lead to formation and/or aggravation of GVs, and a consequent increased risk of bleeding. In fact, previous studies have shown increased portal pressure [23, 24] and GVs development [13] after EVs eradication by EIS. However, because the mechanism of varix obliteration differs between EIS and EBL, it is not clear whether EBL also affects portal pressure and GVs development. Some studies have suggested that EVs eradication by EBL is associated with increased portal pressure and risk of bleeding [25], while other studies have suggested that EBL does not affect portal pressure [26] and that it is not associated with GVs development [20]. In the present study, consistent with that latter finding, GVs did not aggravate after EVs eradication by EBL in all enrolled patients.

The incidence of variceal bleeding in the current study was as low as 9.5% at 3 years after EVs eradication. This result supports the assertion that EVs eradication by EBL prevents bleeding from EVs. As with EVs recurrence, variceal bleeding was significantly more frequent in patients who undergone EBL for secondary prophylaxis than in patients who had undergone EBL for primary prophylaxis. Another independent risk factor for variceal bleeding after EVs eradication in this study was younger age (≤55 years). The underlying cause of liver disease was alcoholic liver disease in about half of enrolled patients; thus, more frequent drinking in younger people, prompted by an increase in social activity, may have caused this result.

This study had some limitations. First, previous studies have suggested that the main pathway for portosystemic shunt is not the EVs, but deeper level varices such as the paraesophageal or periesophageal varices [21], and that the size of these varices, as evaluated using endoscopic ultrasound, is significantly associated with EVs recurrence after EIS or EBL [23, 24]. The results of the present study would be more interesting if such an examination had been performed; as it was, we were not able to evaluate this relationship. Second, because this study was performed retrospectively, several biases may have affected our results. Therefore, further prospective studies are required to confirm our results.

In conclusion, cardiac varices frequently disappear with EVs eradication by EBL; the disappearance of cardiac varices is associated with the size of cardiac varices before EBL, as well as with lower incidence of EVs recurrence after eradication. For this reason, specific therapy for cardiac varices is not required in patients with GOV1s who have been treated using EBL for EVs eradication. However, because persistence of cardiac varices after EVs eradication is associated with the risk of EVs recurrence, more careful surveillance is required in such patients.

## Figures and Tables

**Table 1:** Baseline characteristics of patients who underwent EVs eradication by EBL—arranged according to the type of varices.

	All patients (*n* = 123)	Pts with EVs only (*n* = 38)	Pts with GOV1s (*n* = 85)	*P*
Age	59.7 ± 11.7	60.0 ± 12.3	59.6 ± 11.4	0.347
Male, *n* (%)	96 (78.0)	27 (71.1)	69 (81.2)	0.210
Etiology, *n* (%)				0.158
Hepatitis B virus	41 (33.3)	9 (23.7)	32 (37.6)	
Hepatitis C virus	10 (8.1)	4 (10.5)	6 (7.1)	
Alcohol	59 (48.0)	23 (60.5)	36 (42.4)	
Others	13 (10.6)	2 (5.3)	11 (12.9)	
Purpose of EBL, *n* (%)				0.445
Primary prophylaxis	71 (57.7)	20 (52.6)	51 (60.0)	
Secondary prophylaxis	52 (42.3)	18 (47.4)	34 (40.0)	
Size of EVs				0.639
F2	104 (84.6)	33 (86.8)	71 (83.5)	
F3	19 (15.4)	5 (13.2)	14 (16.5)	
Red color sign on EVs, *n* (%)	117 (95.1)	35 (92.1)	82 (96.5)	0.299
Number of EBL sessions	2.3 ± 1.0	2.1 ± 1.2	2.4 ± 0.8	0.112
Number of used rubber bands	7.7 ± 3.6	7.3 ± 5.0	7.9 ± 2.7	0.456
Duration until EVs eradiation, months	2.4 ± 1.4	2.2 ± 1.7	2.5 ± 1.2	0.288
Platelet count, ×10^9^/L	89.1 ± 45.2	93.0 ± 44.5	87.3 ± 45.6	0.526
INR	1.4 ± 0.3	1.4 ± 0.3	1.4 ± 0.3	0.362
AST, IU/L	89.8 ± 182.7	69.7 ± 59.6	98.8 ± 216.0	0.416
ALT, IU/L	41.9 ± 55.3	34.0 ± 25.1	45.4 ± 64.2	0.295
Bilirubin, mg/dL	1.7 ± 1.4	1.8 ± 1.3	1.7 ± 1.5	0.572
Albumin, g/dL	3.2 ± 0.6	3.1 ± 0.5	3.2 ± 0.6	0.287
Creatinine, mg/dL	1.0 ± 0.9	0.9 ± 0.3	1.0 ± 1.1	0.373
Sodium, mEq/L	137.7 ± 4.7	137.6 ± 4.6	137.7 ± 4.7	0.905
Ascites, *n* (%)	65 (52.8)	49 (57.6)	16 (42.1)	0.111
Encephalopathy, *n* (%)	10 (8.1)	1 (2.6)	9 (10.6)	0.136
Child-Pugh score	7.2 ± 1.7	7.2 ± 1.6	7.2 ± 1.8	0.908
Child-Pugh classification, *n* (%)				0.943
Grade A	50 (40.7)	16 (42.1)	34 (40.0)	
Grade B	61 (49.6)	18 (47.4)	43 (50.6)	
Grade C	12 (9.8)	4 (10.5)	8 (9.4)	

AST: aspartate aminotransferase; ALT: alanine aminotransferase; EBL: endoscopic band ligation; EVs: esophageal varices; GOVs: gastroesophageal varices; INR: international normalized ratio.

**Table 2:** Baseline characteristics of patients with GOV1s—arranged according to the changes in cardiac varices after eradication of esophageal varices by endoscopic band ligation.

	Patients with disappearance of cardiac varices (*n* = 55)	Patients with persistent cardiac varices (*n* = 30)	*P*
Age	60.1 ± 11.0	57.8 ± 12.2	0.295
Male, *n* (%)	44 (80.0)	25 (83.3)	0.707
Etiology, *n* (%)			0.711
Hepatitis B virus	23 (41.8)	9 (30.9)	
Hepatitis C virus	4 (7.3)	2 (6.7)	
Alcohol	21 (38.2)	15 (50.0)	
Others	7 (12.7)	4 (13.3)	
Purpose of EBL, *n* (%)			0.643
Primary prophylaxis	34 (61.8)	17 (56.7)	
Secondary prophylaxis	21 (38.2)	13 (43.3)	
Size of EVs			0.235
F2	44 (80.0)	27 (90.0)	
F3	11 (20.0)	3 (10.0)	
Red color sign on EVs, *n* (%)	54 (98.2)	28 (93.3)	0.247
Size of cardiac varices, cm	1.0 ± 0.2	1.1 ± 0.3	0.046
Number of EBL sessions	2.5 ± 0.9	2.3 ± 0.7	0.226
Number of used rubber bands	8.1 ± 2.8	7.5 ± 2.7	0.342
Duration until EVs eradiation, months	2.7 ± 1.3	2.2 ± 1.0	0.105
Red color sign on cardiac varices, *n* (%)	1 (1.8)	1 (3.3)	0.660
Platelet count, ×10^9^/L	88.7 ± 43.3	84.9 ± 50.3	0.716
INR	1.3 ± 0.2	1.4 ± 0.4	0.265
AST, IU/L	79.0 ± 204.4	135.1 ± 235.0	0.255
ALT, IU/L	36.6 ± 45.6	61.3 ± 87.5	0.157
Bilirubin, mg/dL	1.4 ± 1.1	2.1 ± 1.9	0.075
Albumin, g/dL	3.3 ± 0.6	3.2 ± 0.6	0.557
Creatinine, mg/dL	1.1 ± 1.3	0.9 ± 0.3	0.428
Sodium, mEq/L	137.7 ± 5.1	137.8 ± 4.1	0.971
Ascites, *n* (%)	29 (52.7)	20 (66.7)	0.214
HE, *n* (%)	5 (9.1)	4 (13.3)	0.544
Child-Pugh score	7.1 ± 1.7	7.5 ± 1.9	0.242
Child-Pugh classification, *n* (%)			0.647
Grade A	24 (43.6)	10 (33.3)	
Grade B	26 (47.3)	17 (56.7)	
Grade C	5 (9.1)	3 (10.0)	

AST: aspartate aminotransferase; ALT: alanine aminotransferase; EBL: endoscopic band ligation; EVs: esophageal varices; GOVs: gastroesophageal varices; INR: international normalized ratio.

**Table 3:** Univariate Cox regression analysis for EVs recurrence, variceal bleeding, and mortality after EVs eradication by EBL.

Variable	Rating	*P* value		
		EVs recurrence	EVs bleeding	Mortality
Age	Years	0.183	0.009	0.033
Sex	1 = women; 2 = men	0.449	0.375	0.208
Underlying liver disease	1 = alcohol; 2 = others	0.750	0.343	0.799
Purpose of EBL	1 = primary; 2 = secondary	0.007	0.007	0.881
Type of varices	1 = EVs only; 2 = GOV1s	0.217	0.597	0.679
Size of EVs at baseline	1 = F2; 2 = F3	0.662	0.816	0.261
Red color sign on EVs at baseline	1 = no; 2 = yes	0.766	0.675	0.678
Size of CVs	cm	0.958	0.938	0.118
Duration until EVs eradication	Months	0.584	0.478	0.749
Number of EBL sessions	Number	0.166	0.840	0.233
Number of used rubber bands	Number	0.212	0.928	0.296
CVs disappearance	0 = no CVs; 1 = disappeared CVs; 2 = persistent CVs	0.005	0.106	0.911
Platelet count	×10^9^/L	0.587	0.301	0.293
INR	Ratio	0.366	0.747	0.890
AST, IU/L	IU/L	0.861	0.676	0.275
ALT, IU/L	IU/L	0.466	0.816	0.055
Bilirubin, mg/dL	mg/dL	0.718	0.623	0.089
Albumin, g/dL	g/dL	0.253	0.083	0.201
Creatinine, mg/dL	mg/dL	0.112	0.047	0.297
Sodium, mEq/L	mEq/L	0.980	0.841	0.004
Ascites	1 = no; 2 = yes	0.857	0.967	0.026
Encephalopathy	1 = no; 2 = yes	0.854	0.134	0.003
Child-Pugh score	Score	0.771	0.571	0.008
Child-Pugh classification	1 = A; 2 = B; 3 = C	0.883	0.786	0.164

AST: aspartate aminotransferase; ALT: alanine aminotransferase; CVs: cardiac varices; EBL: endoscopic band ligation; EVs: esophageal varices; GOVs: gastroesophageal varices; INR: international normalized ratio.

## Abbreviations

ALT: Alanine aminotransferase
AST: Aspartate aminotransferase
CI: Confidence interval
EBL: Endoscopic band ligation
EIS: Endoscopic injection sclerotherapy
EVs: Esophageal varices
GOVs: Gastroesophageal varices
GVs: Gastric varices
HR: Hazard ratio
IGVs: Isolated gastric varices
INR: International normalized ratio.
